# Establishment and characterization of patient-derived xenograft models of gastrointestinal stromal tumor resistant to standard tyrosine kinase inhibitors

**DOI:** 10.18632/oncotarget.20816

**Published:** 2017-09-11

**Authors:** Young-Soon Na, Min-Hee Ryu, Changhoon Yoo, Ju-Kyung Lee, Jung Min Park, Chae-Won Lee, Sun Young Lee, Young-Kyoung Shin, Ja-Lok Ku, Sung-Min Ahn, Yoon-Koo Kang

**Affiliations:** ^1^ Asan Institute for Life Science, Asan Medical Center, Seoul, Korea; ^2^ Department of Oncology, Asan Medical Center, University of Ulsan College of Medicine, Seoul, Korea; ^3^ Korean Cell Line Bank, Laboratory of Cell Biology, Cancer Research Institute, Seoul National University College of Medicine, Seoul, Korea; ^4^ Gachon Institute of Genome Medicine and Science, Gachon University Gil Hospital, Incheon, Korea

**Keywords:** gastrointestinal stromal tumor, patient-derived xenograft, KIT mutation, receptor tyrosine kinase inhibitors, resistance

## Abstract

Gastrointestinal stromal tumors (GISTs) with *KIT* or platelet-derived growth factor receptor alpha (*PDGFRa*) oncogenic driver gene mutations, respond to tyrosine kinase inhibitors (TKIs) including imatinib, sunitinib, and regorafenib. However, most patients develop TKI resistance; therefore, novel agents are required. We established three TKI-resistant GIST patient-derived xenograft (PDX) models for effective drug development. These were PDX models harboring primary and secondary *KIT* and additional mutations; *KIT* exon 11 (p.Y570_L576del), *KIT* exon 17 (p.D816E), and *PTEN* (p.T321fs) mutations in GIST-RX1 from a patient who was unresponsive to imatinib, sunitinib, and sorafenib, and *KIT* exon 11 (p.K550_splice) and *KIT* exon 14 (p.T670I) mutations in GIST-RX2 and *KIT* exon 9 (p.502_503insYA) and *KIT* exon 17 (p.D820E) mutations in GIST-RX4 from patients with imatinib and imatinib/sunitinib resistance, respectively. The histological features and mutation statuses of GIST PDXs were consistent with those of the original patient tumors, and the models showed TKI sensitivity comparable to clinical responses. Imatinib inhibited the KIT pathway in imatinib-sensitive GIST-T1 but not GIST-RX1, RX2, and RX4. These GIST PDX models will be useful for studying TKI resistance mechanisms and evaluating novel targeted agents in GIST.

## INTRODUCTION

The gastrointestinal stromal tumor (GIST) is the most common mesenchymal tumor of the gastrointestinal tract. GIST has activating mutations in the *KIT* or platelet-derived growth factor receptor alpha (*PDGFRa*) gene, which act as the main oncogenic drivers [1–3] and are harbored by 75–80% and approximately 10% of tumors, respectively. Tyrosine kinase inhibitors (TKIs) have improved the survival of patients with advanced GISTs, dramatically. Successful clinical trials have led to the approval of imatinib, sunitinib, and regorafenib for treating GISTs. However, most patients eventually develop disease progression with these drugs. Resistance to TKIs is linked to distinctive clinical and molecular features, and the development of secondary mutations in *KIT* or *PDGFRa* is the most common mechanism. Secondary mutations in *KIT*s have been reported in exons 13, 14, 16, 17, and 18 [4]. Inter-/intra-tumoral genetic heterogeneity and clonal evolution with treatment in advanced disease enable multiple secondary mutations to be detected in the same patient [5]. A better understanding of resistance of GISTs to TKIs is necessary to enhance the efficiency of drug development for TKI-refractory GISTs.

The limited availability of preclinical models of GISTs hampers the development of effective therapy, particularly for TKI-resistant GIST. Patient-derived xenografts (PDXs) are useful preclinical models generated by transplanting tumors from human patients into immunocompromised mice. Compared to cell lines and cell line-based xenograft models, PDXs have advantages, in that they reflect real tumor heterogeneity and complexity. PDX models have been established using numerous tumor types. However, only a few GIST PDX models have been reported to date. To understand the mechanisms of TKI resistance and screen potentially effective novel agents for TKI-resistant GISTs, establishing various GIST PDX models is crucial, particularly for TKI-resistant forms.

Based on this background, we generated PDXs from patients with GIST who showed resistance to TKIs. In this report, we present the genetic and phenotypic characteristics of our TKI-resistant GIST PDX models.

## RESULTS

### Establishment of three GIST PDX models

**Figure 1:**
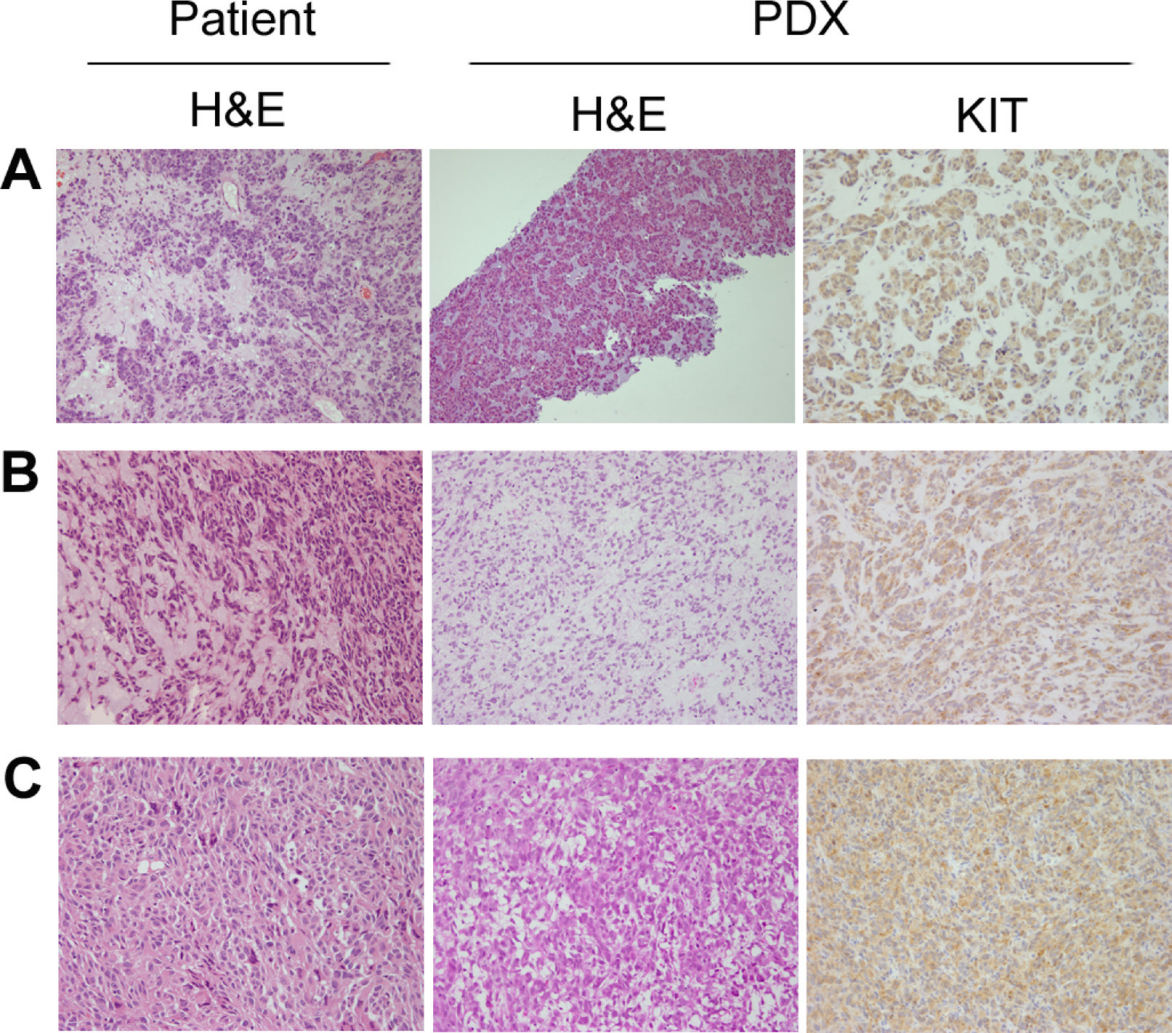
Immunohistochemical (IHC) analysis of original patient tumors and patient-derived xenograft (PDX) models Hematoxylin and eosin (H&E) staining of patient tumor and PDX model samples and KIT immunostaining of tumors of PDX models. (**A**) GIST-RX1. (**B**) GIST-RX2. (**C**) GIST-RX4. 200× magnification.

We established three PDX models from GIST lesions resistant to imatinib, sunitinib, or both (Table 1). Our histological analysis showed that all three models diffusely expressed KIT, a histological hallmark of GIST. We also demonstrated that the original patient and matched established tumors from the PDX models exhibited the same histological features in the H&E staining and KIT IHC images (Figure 1). The histological features of PDX models were consistent throughout passages (Supplementary Figure 1). In addition, the genetic identity of the tumor-PDX pairs was further confirmed using a short tandem repeat (STR) analysis (Supplementary Table 1).

**Table 1:** Clinicopathological characteristics of patients whose samples were used for three gastrointestinal stromal tumor (GIST) patient-derived xenograft (PDX) models

PDX	Patient					
	Age (years)	Sex	Primary site	Resection site	Drug exposure (months)	Best response to TKI
GIST-RX1	67	F	small bowel	peritoneum	imatinib (43)	PR
					sunitinib (9)	SD
					sorafenib (2)	PD
GIST-RX2	42	M	stomach	stomach	imatinib (20)	PR
GIST-RX4	79	M	small bowel	small bowel	imatinib (26)	PD
				liver	sunitinib (5)	SD

TKI, tyrosine kinase inhibitor; PR, partial response; SD, stable disease; PD, progressive disease.

### Mutation analysis of tumor pairs from patients and PDX models

**Figure 2:**
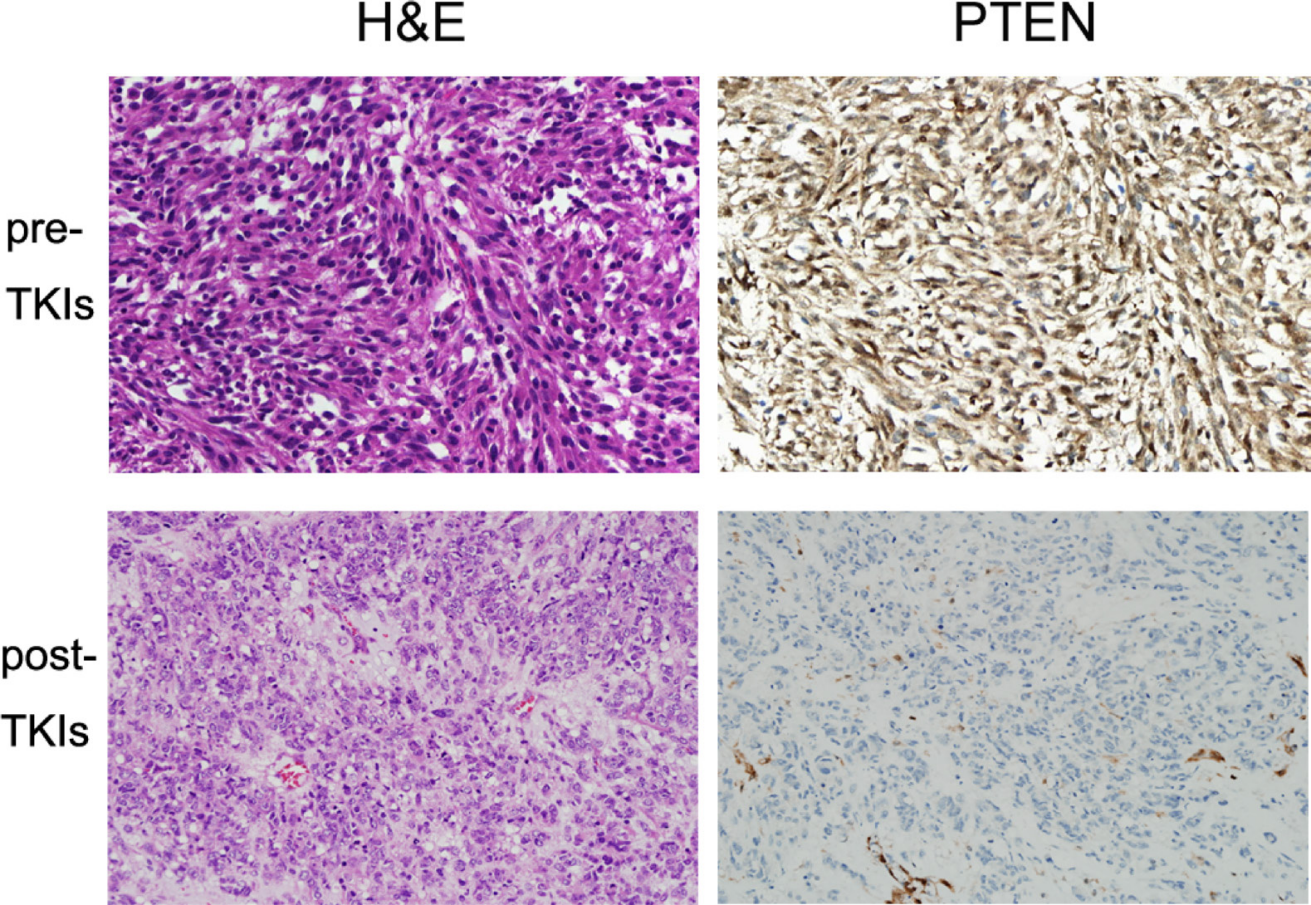
Immunohistochemical (IHC) analysis of hematoxylin and eosin (H&E) staining and phosphatase and tensin homolog (PTEN) in original patient GIST-RX1 samples (upper panel) Tumor obtained from pre-TKI. (lower panel) Tumor obtained from post-TKI which is TKI-resistant. Right bottom shows PTEN-positive endothelial cells of blood vessel and PTEN-negative tumor cells. 200× magnification.

Key mutations from whole exome sequencing (WES) in three patients with GIST were validated in PDX and patient tumors using Sanger sequencing and OncoMap v4. We confirmed that the tumors from the PDX models retained the same set of key mutations identified in the original patient tumors. Table 2 shows the *KIT* and additional somatic mutations. All tumors from the three PDX models had both primary and secondary *KIT* mutations, which explains their resistance to TKIs, i.e., *KIT* exon 11 (p.Y570_L576del) and *KIT* exon 17 (p.D816E) mutations in GIST-RX1, *KIT* exon 11 (p.K550_splice) and *KIT* exon 14 (p.T670I) mutations in GIST-RX2, and *KIT* exon 9 (p.502_503insYA) and *KIT* exon 17 (p.D820E) mutations in GIST-RX4. However, the first biopsy or surgical tumor tissues analyzed at the initial diagnosis harbored only primary *KIT* mutations. GIST-RX1 also harbors a frame-shift mutation in *PTEN* (p.T321fs). The patient tumor tissues prior to the use of TKIs and post-TKIs treatment were immunostained for PTEN. We observed that the patient's tumor post-TKIs, which is the origin of GIST-RX1 and TKI-resistant, was negative for PTEN IHC, whereas the patient's tumor tissue at diagnosis prior to TKIs was positive for PTEN IHC (Figure 2).

**Table 2:** Mutations found in patient-derived xenograft (PDX) models

PDX	*KIT* mutations		Additional mutations
	primary	secondary	
GIST-RX1	p.Y570_L576del (exon11)	p.D816E (exon17)	*PTEN* (p.T321fs)
GIST-RX2	p.K550_splice (exon11)	p.T670I (exon14)	
GIST-RX4	p.502_503insYA (exon9)	p.D820E (exon17)	

PTEN, phosphatase and tensin homolog.

### *In vivo* efficacy testing of imatinib, sunitinib, and regorafenib using GIST PDX models

**Figure 3:**
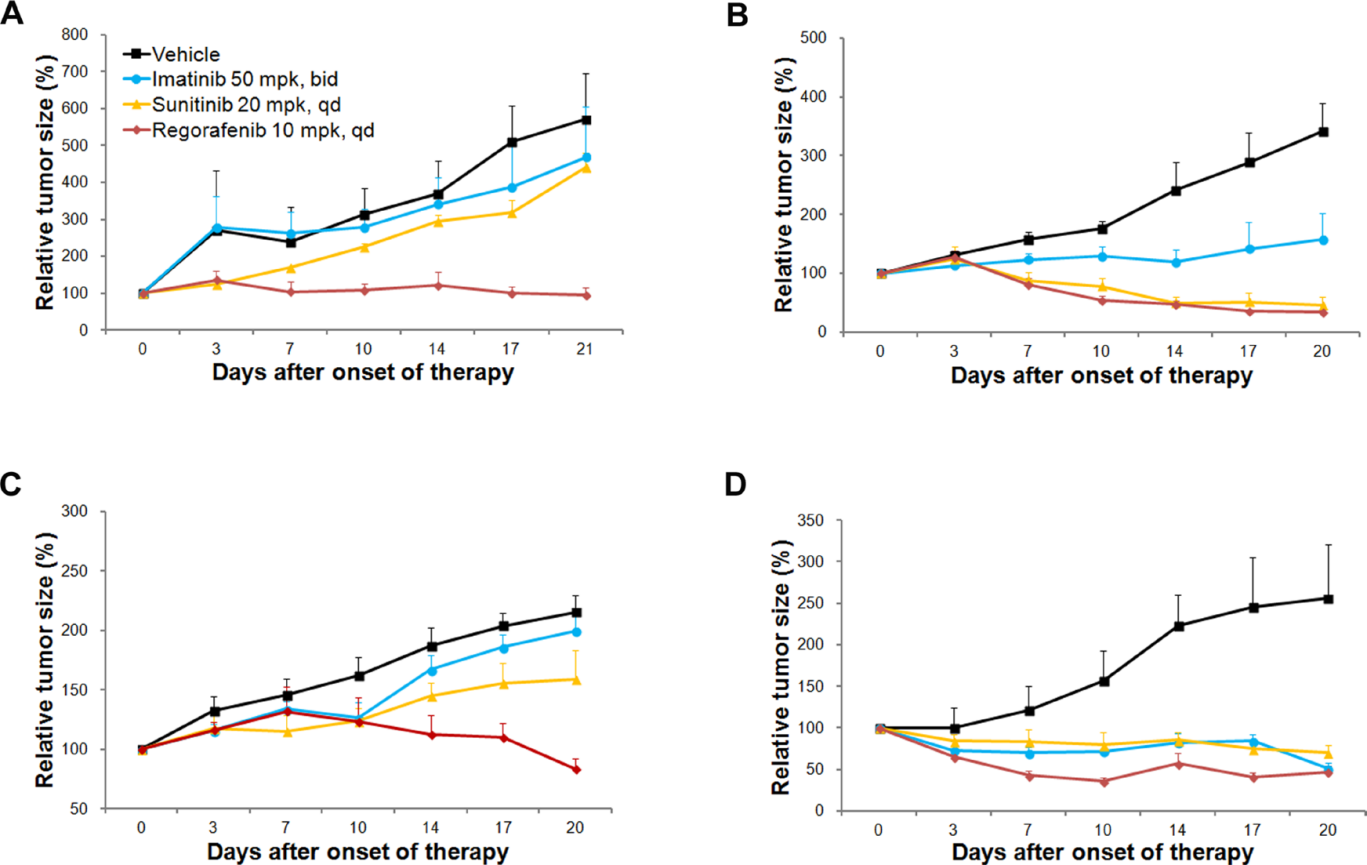
Evaluation of response to receptor tyrosine kinase inhibitors (TKIs) in TKI-resistant gastrointestinal stromal tumor (GIST) patient-derived xenograft (PDX) models and imatinib-sensitive GIST-T1 xenograft models Relative tumor growth was measured. (**A**) GIST-RX1, (**B**) GIST-RX2, (**C**) GIST-RX4, and (**D**) GIST-T1 xenografts. mpk, mg/kg.

We performed *in vivo* efficacy testing of imatinib, sunitinib, and regorafenib using the three GIST PDX models (Figure 3). GIST-RX1 was obtained from a patient whose GIST did not respond to imatinib, sunitinib, and sorafenib; GIST-RX1 responded to regorafenib but not imatinib and sunitinib (Figure 3A). GIST-RX2 was from a patient whose GIST progressed clinically after imatinib treatment, and it was sensitive to sunitinib and regorafenib, but showed only a modest response to imatinib treatment (Figure 3B). GIST-RX4 was from a patient whose GIST did not respond to imatinib and sunitinib and it responded to regorafenib but not imatinib and sunitinib (Figure 3C).

We established a xenograft model using a GIST-T1 cell line, which was sensitive to imatinib. The GIST-T1 xenograft model was sensitive to imatinib, sunitinib, and regorafenib (Figure 3D). As expected, the responses to imatinib in all three established GIST PDX models were lower than the responses in the GIST-T1 xenograft models were, following a 21-day imatinib treatment; The tumor growth was inhibited by 17.8%, 53.9%, and 7.4% in the GIST-RX1, GIST-RX2, and GIST-RX4 models, respectively and by 80.0% in the GIST-T1 xenografts. The GIST-RX1 and GIST-RX4 models showed slight tumor growth inhibition (22.9% and 26.2% decrease in tumor volume) following a 21-day treatment with sunitinib while the GIST-RX2 and GIST-T1 xenografts were highly sensitive to sunitinib (86.7% and 72.5% decrease, respectively).

### Analysis of KIT signaling using receptor TKIs in GIST PDX models

**Figure 4:**
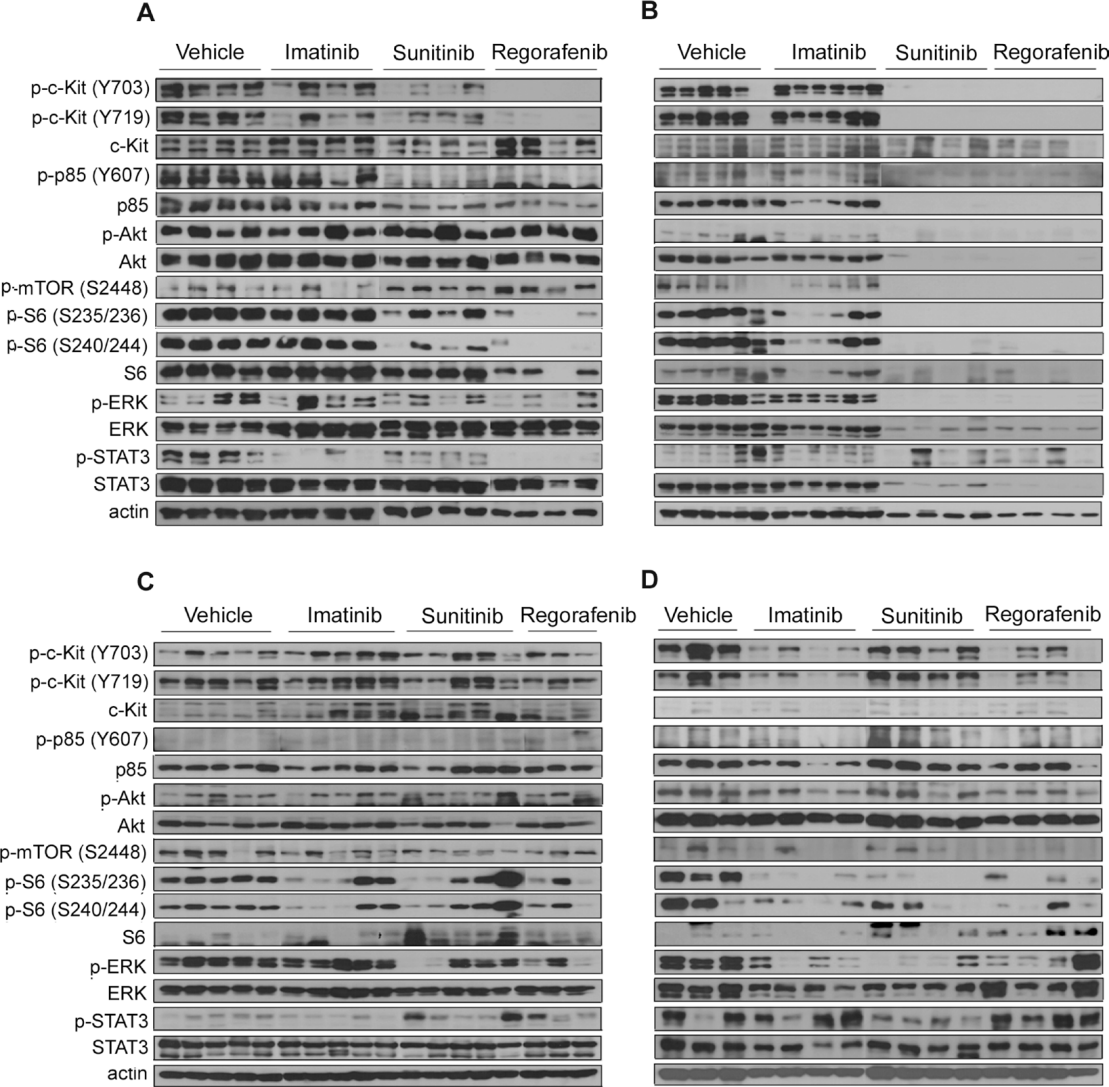
Effects of imatinib, sunitinib, or regorafenib on the expression of KIT signaling-related proteins (**A**) GIST-RX1, (**B**) GIST-RX2, (**C**) GIST-RX4, and (**D**) GIST-T1 xenografts. GIST, gastrointestinal stromal tumor; ERK, extracellular signal-regulated kinase; STAT, signal transducer and activator of transcription 3; mTOR, mechanistic target of rapamycin.

Western blotting of the GIST-RX1 model samples treated with imatinib revealed slight or no inhibition of activated KIT, phosphoinositide-3 kinase (PI3K)/AKT/mTOR, or ERK. The phosphorylation of KIT, ERK, S6, and STAT3 was slightly and markedly inhibited by sunitinib and regorafenib, respectively while phosphorylation of AKT was not inhibited by sunitinib and regorafenib in this model (Figure 4A). Evidence of the lack of inhibition of the activated PI3K pathway by imatinib and the deficiency of PTEN expression due to *PTEN* (p.T321fs) mutation collectively in GIST-RX1 led us to investigate further the antitumor effects of the PI3K inhibitor LY294002 alone or in combination with imatinib in this model (Supplementary Figure 2). In the GIST-RX1 model, the response to LY294002 alone was higher than the response to imatinib alone while the combination of both agents led to higher tumor shrinkage than was shown by either agent alone.

In the GIST-RX2 model, KIT signaling was not inhibited by imatinib at all. Further, pS6 and pERK were not significantly inhibited. In contrast, sunitinib and regorafenib completely inhibited KIT signaling in this model (Figure 4B). In the GIST-RX4 model, we did not observe any significant differences in the phosphorylation levels of KIT, AKT, and mTOR pathway between the vehicle and TKI-treated groups, although some animals showed a reduction in p-S6 and p-ERK following sunitinib and regorafenib treatment (Figure 4C). As expected, GIST-T1 xenograft model showed the inhibition of KIT signaling after imatinib treatment in contrast to three TKI-resistant GIST PDX models. Sunitinib as well as regorafenib inhibited phosphorylation of KIT, and phosphorylation of S6, ERK, and STAT3 in GIST-T1 model (Figure 4D).

## DISCUSSION

We established three GIST PDX models using tumor samples from patients who clinically progressed after treatment with current standard TKIs. In terms of mutation and drug resistance profiles, our PDX models are distinct from previously reported models [6–10]. Our GIST PDX models, GIST-RX1, GIST-RX2, and GIST-RX4, harbored secondary mutations in *KIT* exon 17 (p.D816E), exon 14 (p.T670I), and exon 17 (p.D820E), respectively, which have not been reported in prior studies.

To date, GIST PDX models have only been established in a few studies [6–11]. Among them, only two PDX models had both primary and secondary mutations in *KIT* exons 11 and 17 [6–10]. One model (UZLX-GIST9 with *KIT* p.P577del + W557LfsX5 + D820G mutation) was established from a metastatic lesion that showed clinical progression after imatinib, sunitinib, and regorafenib treatment and the other (PDX with p.Lys558_Glu562del and D816H mutation) was established from a metastatic lesion after the failure of imatinib. Most of the other GIST PDX models were established using tumor tissues not previously exposed to TKIs and harbored primary mutation alone in either the *KIT* exon 9 or *KIT* exon 11. In the previously reported GIST PDX models, drug sensitivity tests of all current standard TKIs were conducted only in UZLX-GIST9, and they showed definite resistance to imatinib, delayed tumor growth with sunitinib, and tumor shrinkage with regorafenib. However, in other PDX models, imatinib inhibited growth or drug sensitivity testing of current standard TKIs was not reported.

In addition to *KIT* mutations, GIST-RX1 has *PTEN* exon 8 (p.T321fs) mutation. *PTEN* (p.T321fs) has been previously reported in the colon, breast, and endometrial cancers [12, 13], but has not yet been found in GIST. This mutation is one of the hotspots of *PTEN* mutations. It is highly probable that *PTEN* (p.T321fs) directly causes resistance to imatinib in GIST. It is well known that diverse *PTEN* missense, nonsense, insertion, and deletion mutations induce loss of PTEN expression and function [14]. In our GIST-RX1 model with *PTEN* (p.T321fs) mutation, GIST tissue obtained at the time of PDX establishment did not express PTEN protein, while the patient's tumor tissue at diagnosis prior to TKIs was positive for PTEN IHC. In the GIST-RX1 model, no expression of PTEN protein indicated the functional loss of PTEN. Theoretically, *PTEN* (p.T321fs) mutation may lead to inadequate PTEN function with inhibition of the phosphorylation of PTEN on Tyr336 by RAK and focal adhesion kinase (FAK), and subsequent cancer progression [15–17]. Moreover, a study in *BRAF*-mutant melanoma suggested that *PTEN* (p.T321fs) mutation may affect PTEN function with *PTEN* (p.T321fs) mutation-inducing resistance to BRAF inhibitors [18]. PTEN deficiency is related to increased sensitivity to PI3K pathway inhibitors [19–21]. In our GIST-RX1 model, the antitumor effect of LY294002 alone or in combination with imatinib was observed. Our observation suggests that *PTEN* mutation or loss of PTEN expression in GIST may be a good target of PI3K pathway inhibitors.

In our GIST PDX models, the antitumor effects of TKIs were mostly consistent with the clinical courses. The GIST-RX1 model harboring *KIT* exon 11 and 17 (p.D816E) mutations established from a patient who showed clinical progression after treatment with imatinib, sunitinib, and sorafenib, showed resistance to imatinib and sunitinib, but modest antitumor effect with regorafenib. In line with our observation in the GIST-RX1 model, *in vitro* tests in previous studies have demonstrated that regorafenib has modest growth inhibitory activity while sorafenib and sunitinib have little effect on GIST cell lines with the *KIT* exon 17 D816E secondary mutation [22, 23].

The GIST-RX2 model that was established from a growing tumor lesion previously treated with imatinib was expected to show resistance to imatinib. In contrast, imatinib showed modest tumor growth inhibition in the GIST-RX2 model. This discrepancy may be explained by coexistence of imatinib-sensitive clones with imatinib-resistant clones in the clinically progressing GIST tumor. The GIST-RX4 model harboring *KIT* exon 9 and 17 (p.D820E) mutations established from a patient who progressed after treatment with imatinib and sunitinib demonstrated drug sensitivity consistent with *in vitro* tests in previously reported studies [22, 23]. Therefore, the GIST-RX4 model was relatively sensitive to regorafenib but resistant to sunitinib.

Previously, KIT signaling after TKI treatment in GIST-T1/816 cells harboring *KIT* exon 11 and 17 (D816E) mutations and GIST-T1/670 harboring *KIT* exon 11 and 14 (T670I) mutations were reported [22]. Imatinib treatment showed little inhibition of p-KIT and p-ERK in GIST-T1/816, and no inhibition of p-KIT and downstream KIT in GIST-T1/670 cells. The little or no inhibition of KIT signaling by imatinib in our GIST-RX1 and GIST-RX2 PDX models is consistent with the findings in GIST-T1/816 and GIST-T1/670 models. In our PDX models, the degree of KIT signaling inhibition by sunitinib and regorafenib was also similar to observations made in the two cell lines above; i.e., it showed slight inhibition of KIT signaling by sunitinib and marked inhibition by regorafenib in the GIST-RX1 and GIST-T1/816 models. In addition, both sunitinib and regorafenib almost completely inhibited KIT signaling in the GIST-RX2 and GIST-T1/670 models. Compared to the GIST-T1/816 cells, no TKI inhibited p-AKT in our GIST-RX1 model, which was probably caused by loss of PTEN through *PTEN* mutation. In some western blot analyses of the GIST-RX4 model, sunitinib and regorafenib treatments did not significantly inhibit p-mTOR but inhibited p-S6. The regulation of S6K by 3-phosphoinositide dependent protein kinase-1 (PDK-1) may explain the difference in inhibition of p-mTOR and p-S6 status as demonstrated after rapamycin treatment in urothelial carcinoma [24, 25].

In conclusion, we have established and characterized three GIST PDX models harboring distinctive primary and secondary *KIT* mutations that are resistant to imatinib, sunitinib, or both. It is envisaged that the established GIST PDX models will play an important role in developing novel therapeutic agents and elucidating resistance mechanisms in TKI-resistant GIST.

## MATERIALS AND METHODS

### Establishment of TKI-resistant GIST PDX models

Patient-derived GIST xenografts were established from tumors of patients with metastatic GISTs after failure to respond to at least imatinib, sunitinib, or both in nonobese diabetic/severe combined immunodeficiency (NOD-SCID) mice according to a previous report [26]. Tumor and peripheral blood samples were collected from patients who provided written informed consent. This study was approved by the Institutional Review Board and the Institutional Animal Care and Use Committee of Asan Medical Center, Seoul, Korea. The clinicopathological characteristics of three cases are also summarized in Table 1.

In Case 1, the patient had previously undergone surgical resectioning of a localized GIST originating from the small bowel, and 2 years later, the patient developed multiple hepatic and peritoneal metastases and received first-line imatinib for 43 months, second-line sunitinib for 9 months, and third-line sorafenib for 2 months. The best responses to each treatment were a partial response (PR), stable disease (SD), and progressive disease (PD), respectively. In this case, the growing peritoneal metastases were resected 1 month after the third-line sorafenib treatment failed and used for establishment of PDX GIST-RX1.

In Case 2, the patient had gastric GIST with concurrent multiple hepatic metastases at diagnosis and achieved a PR with imatinib. However, the primary gastric GIST lesion definitely increased after 20 months of imatinib treatment while the hepatic metastases were still responsive to imatinib. The growing gastric tumor lesion was resected and subsequently used to establish the PDX GIST-RX2.

In Case 3, the patient received first-line imatinib without evaluable disease after debulking operation of a small bowel GIST and multiple peritoneal metastases at diagnosis. After a 22-month imatinib treatment, new hepatic metastases developed. The patient subsequently received sequentially escalated dose of imatinib and sunitinib for 4 and 6 months, respectively, and then an imatinib rechallenge for 3 months. The best response to each subsequent treatment was SD, SD, and PD, respectively. The growing hepatic metastases after the imatinib rechallenge were resected and subsequently used for PDX GIST-RX4 establishment.

Resected GIST lesions were immediately stored in a chilled medium, and the tumors were diced into 2- to 3-mm pieces and subcutaneously (sc) transplanted into both flanks of 6- to 10-week-old SCID mice. Tumors from the SCID mice were minced under sterile conditions, transplanted into successive BALB/c nude mice, and were passaged up to 10 times.

### Short tandem repeat analysis

Genomic DNAs from tumors of patients and PDXs (passage 3–4) were amplified at loci containing the highly polymorphic microsatellite markers D1S1586 and D3S1765. Polymerase chain reaction (PCR) products were denatured using 95% formamide and electrophoresed on a sequencing gel for 2 hours at a constant power of 60 W, following which the gels were dried and visualized autoradiographically. The DNA was also amplified using an AmpFlSTR identifiler PCR amplification kit (Applied Biosystems, Foster City, CA, USA). PCR was used to amplify 15 tetranucleotide repeat loci and sex-determining markers at loci containing highly polymorphic microsatellite markers. The amplified products were analyzed using an ABI 3730 genetic analyzer (Applied Biosystems, Foster City, CA, USA) [27, 28].

### Immunohistochemistry and hematoxylin-eosin staining

Formalin-fixed, paraffin-embedded, 4-μm tumor sections were dewaxed in xylene, rehydrated with graded alcohol concentrations, and placed in an endogenous peroxide blocking buffer for 15 minutes. Sections were washed in water, antigen-retrieved, and then placed in citrate buffer. Nonreactive staining was blocked by treating the sections with 1% horse serum in Tris-buffered saline (pH 6.0) for 3 minutes. Then, anti-KIT (1:800, A4502, DAKO, Carpinteria, CA, USA) and anti-phosphatase and tensin homolog (PTEN, 1:100, ab32199; Abcam, Cambridge, MA, USA) antibodies were then applied and the antibody binding was detected using an avidin-biotin peroxidase complex (Universal Elite ABC kit, Vectastain, Burlingame, CA, USA) for 10 minutes. Diaminobenzidine tetrahydrochloride solution (Kit HK153-5K; Biogenex, San Ramon, CA, USA) was then used as a chromogen. The tumors specimens were stained with hematoxylin and eosin (H&E) to examine the basic histomorphological features.

### Whole exome sequencing, Sanger sequencing, and OncoMapping

Genomic DNA samples were extracted from tumors using a DNeasy blood and tissue kit (Qiagen, Hilden, Germany) according to the manufacturer's instructions. The quality of DNA was checked by agarose gel electrophoresis and A260/A280 ratios using NanoDrop spectrophotometer (Thermo Scientific, Wilmington, DE, USA). For the GIST-RX2 and GIST-RX4 samples, matched control DNA obtained from the peripheral blood of the patients was sequenced in parallel. The patient's tumor GIST-RX1 DNA was compared with the collective blood DNA of five randomly selected patients with GIST as the normal DNA because no control blood DNA was obtainable from the original patient. Exome enrichment was performed using the TruSeq sample prep (Illumina, San Diego, CA, USA). Sequencing was performed using a HiSeq 2000 (Illumina, San Diego, CA, USA). The average sequencing quality and coverage depth were 35.78 (Phred score) and 92.1x, respectively. Sequenced reads were aligned to the human reference genome hg19 using the Burrows-Wheeler Alignment (BWA) tool [29]. Mutations were identified using Mutect [30] and Genome Analysis Toolkit (GATK) [31] Somatic Indel detector. We annotated mutations using Oncotator [32] and filtered common variants using common-dbsnp build 137 [33]. In this study, we selected splicing site mutations, frameshift insertion/deletions, non-frameshift insertion/deletions, missense mutations, and nonsense mutations that changed the amino acid sequence. Variants in candidate genes were confirmed using Sanger sequencing (DNA link Inc. and Macrogen Inc., Seoul, Korea) in tumors of patients and PDXs. In addition, the mutation analyses of patient tumors and PDXs were validated using the OncoMap v4, a mass spectrometry based genotyping panel, which includes 440 sites in 41 tumor-related genes such as *KIT*, *PDGFRa*, tumor protein 53 (*TP53*), epidermal growth factor receptor (*EGFR*), and B-Raf proto-oncogene (*BRAF*).

### Drug sensitivity test

PDX models were established by transplanting (sc) 2–3 tumor pieces into the left flank of 6-week-old male athymic nude (nu/nu) mice. When the sc tumors reached a size of 200 mm^3^ (day 0), three TKI-resistant PDX or xenograft models established using imatinib-sensitive GIST-T1 cell lines were randomly allocated to one of the following treatment groups: Control (vehicle treated, DW or imatinib, citrate-buffered (pH 3.5) solution for sunitinib, and polyethylene glycol 400 (PEG400)/125 mM aqueous methanesulfonic acid (80/20) for regorafenib), imatinib, sunitinib, and regorafenib. These compounds were purchased from SelleckChem (Houston, TX, USA). Imatinib (50 mg/kg, twice daily [bid]), sunitinib (20 mg/kg, daily [qd]), regorafenib (10 mg/kg, qd), and the vehicle were administered orally (po) for 21 days. LY294002 (50 mg/kg per each dose) purchased from SelleckChem (Houston, TX, USA) was administered intraperitoneally (ip) twice a week. Tumors were measured using a caliper twice weekly and calculated as volume (mm^3^), (length × width^2^)/2 and the body weights were also monitored. The GIST-T1 cell line for the imatinib-sensitive model was obtained from Cosmo Bio (Tokyo, Japan).

### Western blotting

Western blotting was performed using tumors harvested 4 hours post TKI dosing on the last day of drug administration. Dissected frozen tumors were homogenized using TissueLyser II (Qiagen, Hilden, Germany) in T-PER tissue protein extraction reagent (Thermo Scientific, Rockford, IL, USA) with a protease inhibitor cocktail tablet (Roche, Indianapolis, IN, USA) and phosphatase inhibitors (Sigma, St. Louis, MO, USA). Homogenates were centrifuged and analyzed using western blotting. For the KIT signaling pathway analysis, antibodies against phosphorylated p-KIT^Y719^, p-KIT^Y703^, KIT, p-p85^Y607^, p85, p-AKT, AKT, p-mechanistic target of rapamycin (mTOR)^S2448^, p-extracellular signal-regulated kinase (ERK), ERK p-S6^S235/236^, p-S6^S240/244^, S6, p-signal transducer and activator of transcription 3 (STAT3), and STAT3 were obtained from Cell Signaling Technology (Danvers, MA, USA). The antibody against actin was obtained from Sigma-Aldrich (St. Louis, MO, USA). Secondary antibodies conjugated with horseradish peroxidase were obtained from Jackson ImmunoResearch Laboratories (West Grove, PA, USA).

## SUPPLEMENTARY MATERIALS FIGURES AND TABLE


