# Supplementary material for: Establishment and characterization of patient-derived xenograft models of gastrointestinal stromal tumor resistant to standard tyrosine kinase inhibitors
Source: Oncotarget. 2017 Sep 11;8(44):76712–21. doi: 10.18632/oncotarget.20816 (PMC5652737; doi:10.18632/oncotarget.20816)
Supplement: Supplementary file 1 [file oncotarget-08-76712-s001.pdf]

Establishment and characterization of patient-derived xenograft models of gastrointestinal stromal tumor resistant to standard tyrosine kinase inhibitors

SUPPLEMENTARY MATERIALS

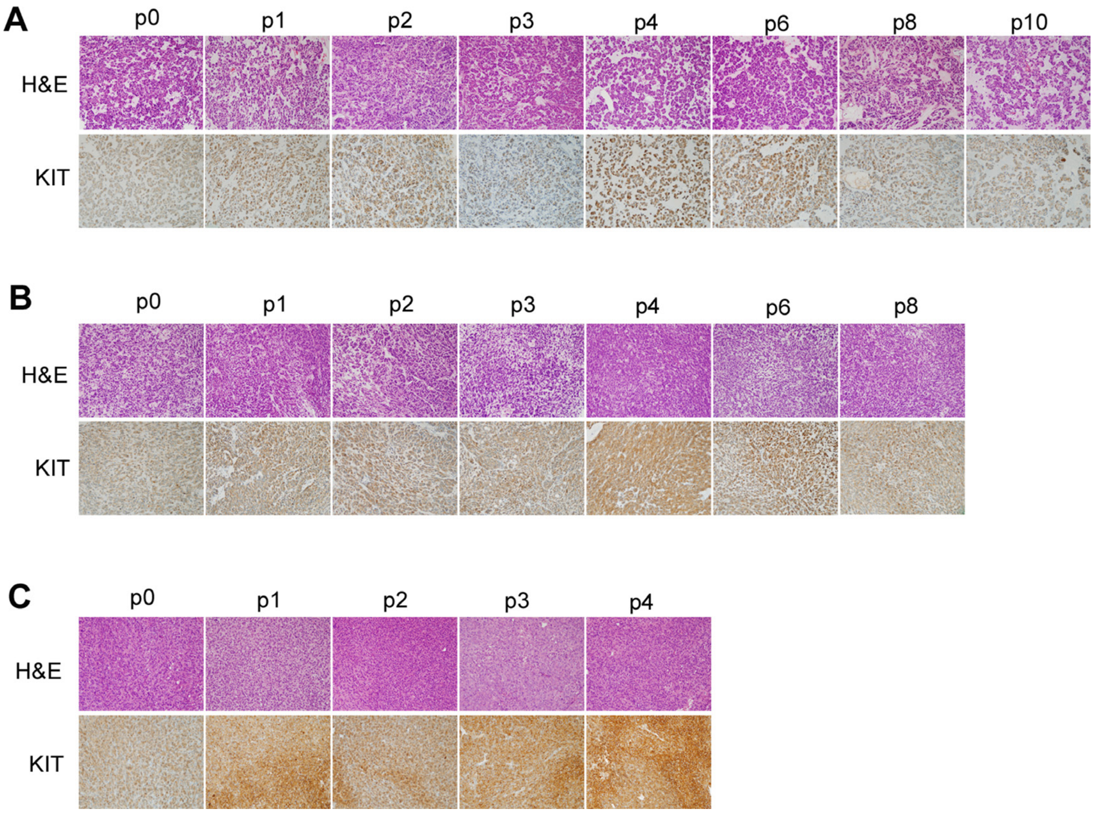

Supplementary Figure 1: The histological features of patient-derived xenograft (PDX) models throughout the passage. Hematoxylin and eosin (H&E) staining and KIT immunostaining of tumors of PDX models. (**A**) GIST-RX1 (epithelioid type). (**B**) GIST-RX2 (mixed type). (**C**) GIST-RX4 (epithelioid type). p, passage; 200× magnification.

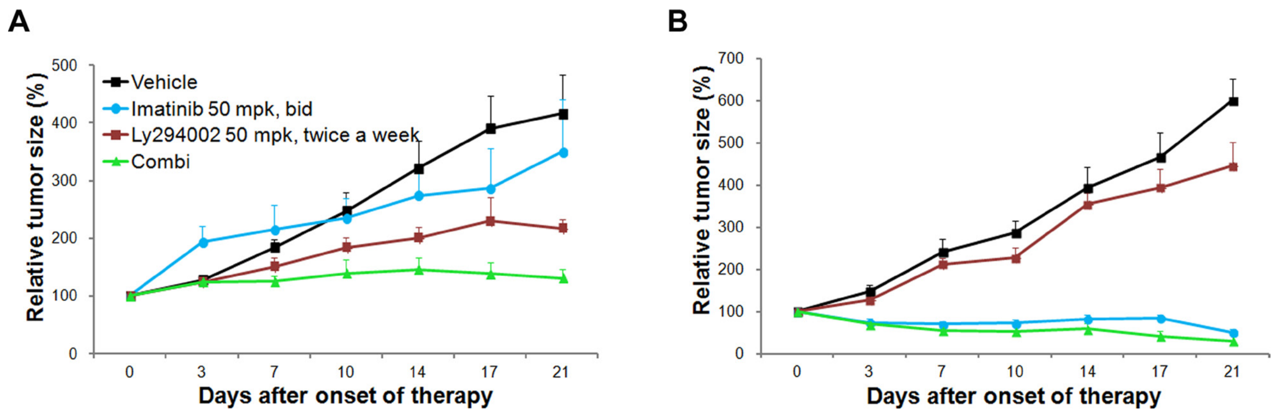

Supplementary Figure 2: (**A**) Antitumor effects of phosphoinositide-3 kinase (PI3K) inhibitor LY294002 alone or combination with imatinib in GIST-RX1 model and (**B**) gastrointestinal stromal tumor (GIST)-T1 xenografts. Relative tumor growth was measured.

Supplementary Table 1: Analysis of short tandem repeats in original patient and patient-derived xenograft (PDX) model tumors

Sample	D8S1179	D21S11	D7S820	CSF1PO	D3S1358	TH01	D13S317	D16S539
original patient tumor	11,16	29,30	10,11	10,11	16	6,9.3	8	OL,12
GIST-RX1	11,16	29,30	10,11	10,11	16	6,9.3	8	OL,12
original patient tumor	14,15	29,30	9,10	10,12	16,18	7,9	8,11	9,10
GIST-RX2	14,15	29,30	9,10	10,12	16,18	7	8,11	9,10
original patient tumor	11,12	30,32.2	9,11	11,12	15,18	6,9	8,11	11,12
GIST-RX4	11,12	30,32.2	9,11	11,12	15,18	6	11	11,12

Sample	D2S1338	D19S433	Vwa	TPOX	D18S51	Amelogenin	D5S818	FGA
original patient tumor	19,22	13,14	16,18	OL	15,18	X	11,13	OL
GIST-RX1	19,22	13,14	16,18	OL	15,18	X	11,13	17.2,OL
original patient tumor	19,23	13,14.2	14	11	13,16	X,Y	9,12	18,22
GIST-RX2	19,23	13,14.2	14	11	13,16	X,Y	9,12	18
original patient tumor	18,19	13,14.2	16	8	18,21	X,Y	11,12	22,23
GIST-RX4	18,19	13,14.2	16	8	18	X,Y	11,12	22,23
